# Prognostic significance of Wilms’ tumor gene 1 expression in children with B-cell precursor acute lymphoblastic leukemia

**DOI:** 10.3389/fonc.2023.1297870

**Published:** 2024-01-15

**Authors:** Yu-juan Xue, Yu Wang, Le-ping Zhang, Ai-dong Lu, Yue-ping Jia, Ying-xi Zuo, Hui-min Zeng

**Affiliations:** Department of Pediatrics, Peking University People’s Hospital, Peking University, Beijing, China

**Keywords:** B cell precursor acute lymphoblastic leukemia, pediatric, WT1 transcript levels, clinical characteristics, prognosis

## Abstract

**Introduction:**

The prognostic role of Wilms’ tumor 1 (WT1) gene expression at diagnosis in children with B cell precursor acute lymphoblastic leukemia (BCP-ALL) is still controversial.

**Methods:**

We detected the WT1 transcript levels of 533 *de novo* pediatric BCP-ALL patients using TaqMan-based real-time quantitative PCR and analyzed their clinical features.

**Results:**

The WT1 transcript levels differed among the distinct molecularly defined groups, with the highest levels in the *KMT2A* rearrangements (*KMT2A-r*) group. According to the results of the X-tile software, all patients were divided into two groups: WT1/ABL ≥ 0.24% (group A) and <0.24% (group B). The proportions of patients whose age was ≥10 years old, with immunophenotype of Pro-B, belonging in high-risk group, or with minimal residual disease (MRD) ≥ 0.01% at week 12 were significantly higher in group A than in group B. In the B-other group, WT1 overexpression was an independent risk factor of overall survival (OS) rate (*P* = 0.042), and higher MRD ≥ 0.01% at week 12 was associated with lower OS rate (*P*<0.001) and event-free survival rate (*P*<0.001). Moreover, the subgroup analysis revealed that, in patients with initial WBC<50 × 10^9^/L or MRD<0.1% at day 33 or MRD<0.01% at week 12 or in the standard-risk group, WT1 overexpression led to a poorer outcome in comparison with those with WT1 downexpression (*P*<0.05).

**Discussion:**

Therefore, pediatric BCP-ALL with WT1 overexpression had unique clinico-pathological characteristics and poor treatment response. In B-other patients, WT1 overexpression at diagnosis predicted an inferior prognosis. The WT1 gene may serve as a biomarker for monitoring residual disease in the B-other population, especially in children in the standard-risk group.

## Introduction

Acute lymphoblastic leukemia (ALL) is the most common childhood malignancy, originating from lymphatic stem cell progenitors, and B-cell precursor acute lymphoblastic leukemia (BCP-ALL) accounts for approximately 80% of childhood ALL (1). Recently, the 5-year overall survival (OS) rate of pediatric BCP-ALL in developed countries has approached 90% due to the development of a risk-guided treatment system and the progress of therapeutic techniques (2). Molecular genetic abnormalities at initial diagnosis, such as *ETV6-RUNX1*, *TCF3-PBX1*, *BCR-ABL1*, and *KMT2A* rearrangements (*KMT2A-r*), and MRD at various stages of treatment are the most crucial indicators to determine risk stratification and prognosis (2). However, for other BCP-ALL (B-other) populations with no predictive molecular genetic abnormalities, it is necessary to identify new genetic markers to further improve the risk stratification system and select the most appropriate clinical therapeutic schedules for these patients.

Located on human chromosome 11p13, the Wilms tumor 1 (WT1) gene is primarily expressed in normal tissues such as the kidney, reproductive system, and hematological system. It encodes a transcription factor and is involved in biological processes such as embryonic development, cell proliferation, differentiation, apoptosis, invasion, and metastasis (3). WT1 is typically overexpressed in individuals with acute leukemia and expressed at low levels in healthy hematopoietic stem/progenitor cells (4). Although many studies have explored the prognostic value of WT1 expression in acute myeloid leukemia (AML) (5, 6), there is still a lack of research on the prognostic significance of WT1 expression in ALL, particularly in pediatric BCP-ALL (7). Therefore, it is necessary to conduct comprehensive investigations in children with BCP-ALL.

In this study, we retrospectively analyzed the data of 533 children with BCP-ALL admitted to our center, with the aim to explore the prognostic significance of WT1 expression and its correlation with other clinical characteristics in these children.

## Materials and methods

### Patients

From January 2012 to December 2018, 533 consecutive children (aged 0 to 18) newly diagnosed with *de novo* BCP-ALL were included.

### Diagnosis

The diagnosis of BCP-ALL was based on the morphological, immunophenotypic, cytogenetic, and molecular criteria as measured by standard techniques.

The risk stratification was assessed according to NCI risk criteria, cytogenetic subtypes, and treatment response (8).

### Detection of WT1 transcripts and MRD

Real-time quantitative PCR (RQ-PCR), based on TaqMan, was used to detect the quantities of WT1 transcripts at diagnosis as well as *TCF3-PBX1*, *ETV6-RUNX1*, *BCR-ABL1*, and *KMT2A-r* fusion transcripts, which had been described in our previous studies (3).

The WT1 transcript level was ultimately calculated as the ratio of WT1 transcript copies to ABL copies using the control gene Abelson (ABL) as a reference. MRD was detected by multi-parameter flow cytometry with a sensitivity of 0.01% (9).

### Treatment

All patients received a modified version of the Berlin–Frankfurt–Munster regimen. The entire treatment cycle consisted of induction therapy, consolidation therapy, reinduction therapy interspersed with consolidation therapy, and maintenance therapy. The details of the protocol are presented in our previous reports (8, 10).

### Definitions

The B-other group was defined as BCP-ALL children with no *TCF3-PBX1*, *ETV6-RUNX1*, *BCR-ABL1*, or *KMT2A-r*. Complete remission (CR) was defined as the presence of normal hematopoiesis with less than 5% bone marrow blast cells and the absence of an extramedullary disease. OS and event-free survival (EFS) were measured from the date of diagnosis. The endpoint events of EFS were the first relapse or death while in remission, and the endpoint event of the OS was death regardless of the cause. The last follow-up date was June 30, 2022.

### Statistical analysis

All data were analyzed by using SPSS 26.0 (SPSS Inc., Chicago, IL, USA). Continuous variables were compared using Mann–Whitney *U*-test, and categorical variables were compared using the chi-square test or Fisher exact test. OS and EFS were estimated using the Kaplan–Meier method and compared using the log-rank test. Potential prognostic factors were considered in a Cox proportional hazards regression model in multivariate analyses. X-tile 3.6.1 software (Yale University, New Haven, CT, USA) was used to analyze the optimal cutoff value for the WT1 transcript level. According to the results of the X-tile software, the optimal cutoff value for the WT1 transcript level was 0.24% in children with BCP-ALL (**Supplementary Figure S1**). A two-sided *P* < 0.05 was considered statistically significant.

## Results

### Patients’ outcomes

A total of 533 children with BCP-ALL were enrolled in the study, of which 302 were male (**Table 1**). The median age at diagnosis was 5.0 years (range, 0.3–16.0 years).

**Table 1:** Association of WT1 transcript levels with clinical features and treatment response of children with BCP-ALL.

Variables	*N* (%)	WT1/ABL ≥ 0.24% Group A, *N* (%)	WT1/ABL < 0.24% Group B, *N* (%)	*X* ^2^	*P*
All	533 (100)	276 (100)	257 (100)		
Sex				0.080	0.777
Male	302 (56.7)	158 (57.2)	144 (56.0)		
Female	231 (43.3)	118 (42.8)	113 (44.0)		
Age (years)					
<10	387 (72.6)	181 (65.6)	206 (80.2)	14.216	<0.001
≥10	146 (24.7)	95 (34.4)	51 (19.8)		
Initial WBC (×10^9^/L)					
<50	460 (86.3)	231 (83.7)	229 (89.1)	3.294	0.070
≥50	73 (13.7)	45 (16.3)	28 (10.9)		
Immunophenotype					
Pro-B	36 (6.8)	30 (10.9)	6 (2.3)	15.393	<0.001
Common-B	427 (80.1)	207 (75.0)	220 (85.6)	9.391	0.002
Pre-B	70 (13.1)	39 (14.1)	31 (12.1)	0.499	0.480
Genotype					
*ETV6-RUNX1*	83 (15.6)	36 (13.0)	47 (18.3)	2.784	0.095
*KMT2A-r*	25 (4.7)	21 (7.6)	4 (1.6)	10.904	0.001
*TCF3-PBX1*	23 (4.3)	7 (2.5)	16 (6.2)	4.387	0.036
*BCR-ABL1*	27 (5.1)	14 (5.1)	13 (5.1)	0.000	0.994
B-other	375 (70.4)	198 (71.7)	177 (68.9)	0.525	0.469
Risk stratification					
SR	185 (34.7)	85 (30.8)	100 (38.9)	3.866	0.049
IR	248 (46.5)	129 (46.7)	119 (46.3)	0.010	0.920
HR	100 (18.8)	62 (22.5)	38 (14.8)	5.147	0.023
Day 33 MRD				2.370	0.124
<0.1%	443 (83.4)	222 (81.0)	221 (86.0)		
≥0.1%	88 (16.6)	52 (19.0)	36 (14.0)		
Week 12 MRD				4.701	0.030
<0.01%	488 (91.9)	245 (89.4)	243 (94.6)		
≥0.01%	43 (8.1)	29 (10.6)	14 (5.4)		

MRD, minimal residual disease; SR, standard risk; IR, intermediate risk; HR, high risk; WBC, white blood count.

After induction therapy, 527 (99.2%) patients achieved CR. In total, 82 children eventually relapsed, with a median relapse time of 19.2 months (range, 2.8–68.5 months), and 59 children died, including 49 deaths from relapse.

The 5-year OS rate and EFS rate of the entire cohort were 89.2 ± 1.4% and 83.2 ± 1.6%, respectively, with a median follow-up time of 64.6 months (range, 0.8–126.7 months).

### WT1 expression patterns in different molecular subgroups

**Figure 1:**
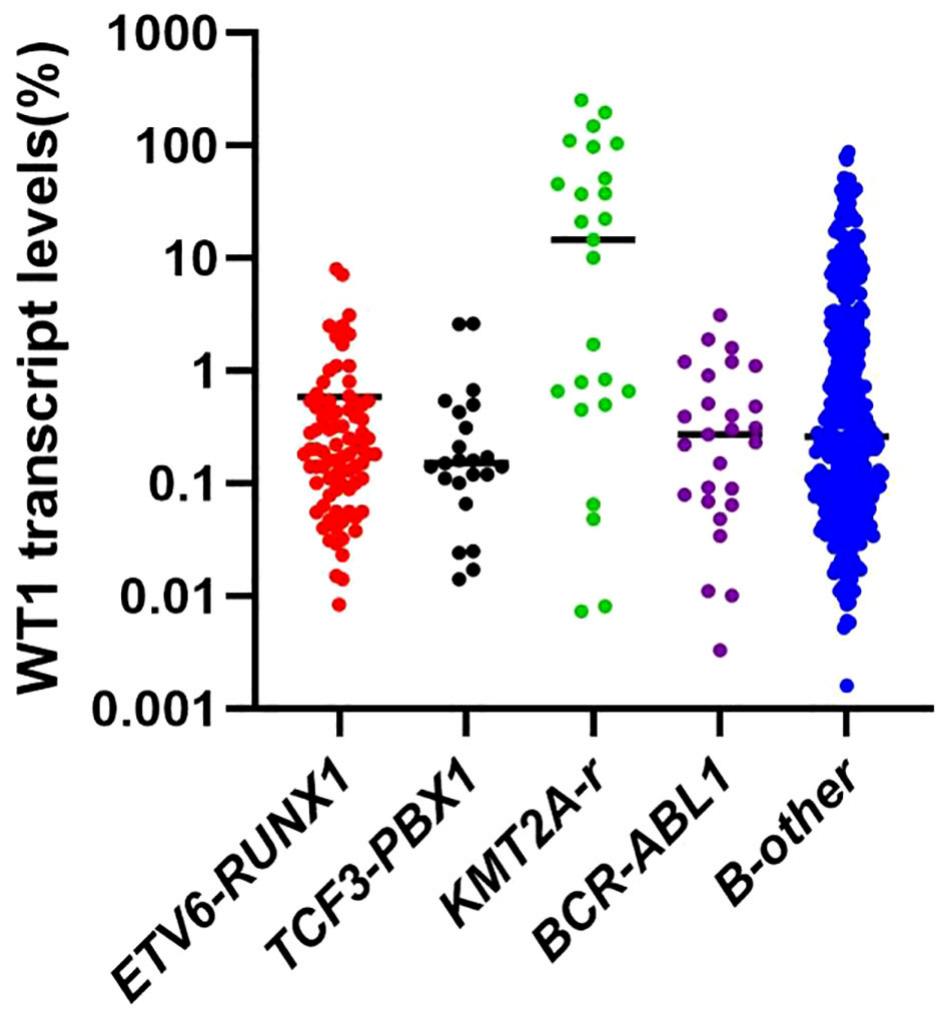
WT1 transcript levels in the different subgroups of children with BCP-ALL.

The median WT1 transcript level at diagnosis in the whole population was 0.26% (range, 0–251.2%). The median WT1 transcript levels in *ETV6-RUNX1*, *TCF3-PBX1*, *KMT2A-r*, *BCR-ABL1*, and B-other groups were 0.19% (range, 0.01–8.0%), 0.15% (range, 0.01–2.62%), 14.5% (range, 0.01–251.19%), 0.27% (range, 0–3.1%), and 0.26% (range, 0–87.7%), respectively (*P* < 0.001). The WT1 transcript level in the *KMT2A-r* group was significantly higher than that in the other groups (**Figure 1**).

### Association of WT1 transcript levels at diagnosis with other clinical features and treatment response

According to the results of the X-tile software, the patients were divided into two groups: WT1/ABL ≥ 0.24% (group A) and < 0.24% (group B). The rates of age ≥10 years old, immunophenotype of Pro-B, genotype of *KMT2A-r* and high-risk patients in group A were significantly higher than those in group B (*P* < 0.05). In group A, patients with MRD ≥ 0.01% at week 12 accounted for 10.6%, which was significantly higher than that in group B (*P* < 0.05). However, the level of WT1 transcript was not associated with sex, initial white blood count, or MRD levels on day 33. The clinical features and treatment response of patients with BCP-ALL with different WT1 transcript levels are summarized in **Table 1**.

### Effects of WT1 overexpression on OS and EFS in the BCP-ALL patients and B-other ALL patients

In the entire cohort of BCP-ALL, patients in group A (with WT1 overexpression) had inferior 5-year OS and EFS rates compared to those in group B (OS: 80.3 ± 4.7% vs. 90.5 ± 1.4%, *P* = 0.008; EFS: 79.8 ± 2.4% vs. 86.9 ± 2.1%, *P* = 0.021). However, multivariate analysis revealed that WT1 overexpression was not an independent risk factor of OS and EFS rate (**Supplementary Table S1**). We further analyzed the impact of WT1 overexpression in the B-other group. Patients with WT1 overexpression had significantly lower 5-year OS and EFS rates than patients with WT1 downexpression (OS: 73.8 ± 6.8% vs. 88.4 ± 1.8%, *P* = 0.007; EFS: 67.8 ± 7.5% vs. 82.1 ± 2.1%, *P* = 0.018). Multivariate analysis revealed that WT1 overexpression was an independent risk factor of OS rate, and a higher MRD level at week 12 was associated with lower OS and EFS rates (**Table 2**).

**Table 2:** Univariate and multivariate analysis of risk factors for overall survival and event-free survival in the B-other ALL patients.

Variable	OS		EFS	
	Univariate (*P*)	Multivariate (*P*) HR (95%CI)	Univariate (*P*)	Multivariate (*P*) HR (95%CI)
Gender	0.868		0.525	
Age (≥10), years	0.012	0.050 1.649 (0.913–2.981)	0.010	0.171 1.260 (0.763–2.081)
WBC ≥ 50 × 10^9^/L	0.034	0.228 1.292 (0.692–2.413)	0.022	0.127 1.519 (0.837–2.755)
Immunophenotype	0.847		0.423	
WT1-overexpression	0.007	0.042 2.024 (1.025–4.000)	0.018	0.063 1.478 (0.908–2.406)
Day 33 MRD ≥ 0.1%	<0.001	0.295 1.524 (0.745–3.116)	<0.001	0.101 1.599 (0.884–2.892)
Week12 MRD ≥ 0.01%	<0.001	<0.001 4.249 (2.294–7.871)	<0.001	<0.001 5.039 (3.061–8.295)

MRD, minimal residual disease; WBC, white blood count; HR, hazards ratio; CI, confidence interval; EFS, event-free survival; OS, overall survival.

### Significance of WT1 expression in different clinical subgroups

**Figure 2:**
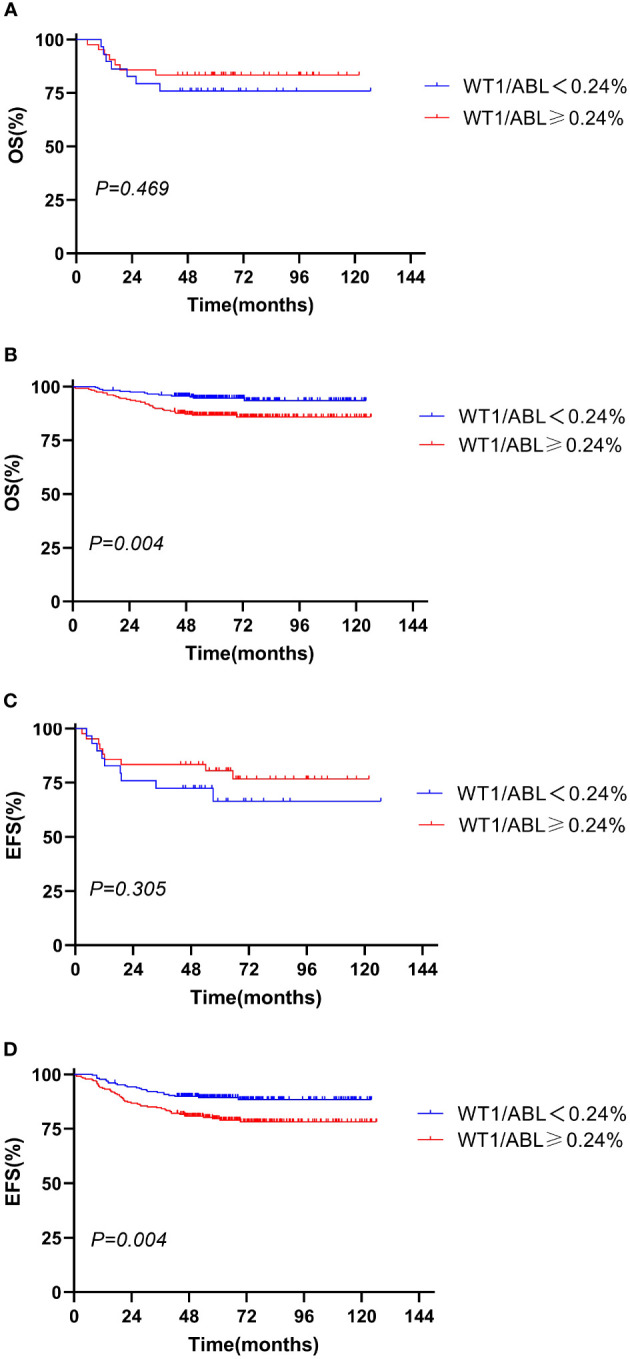
Overall survival and event-free survival according to the WT1 expression and initial WBC. **(A, C)** WBC ≥ 50 × 10^9^/L; **(B, D)** WBC < 50 × 10^9^/L).

**Figure 3:**
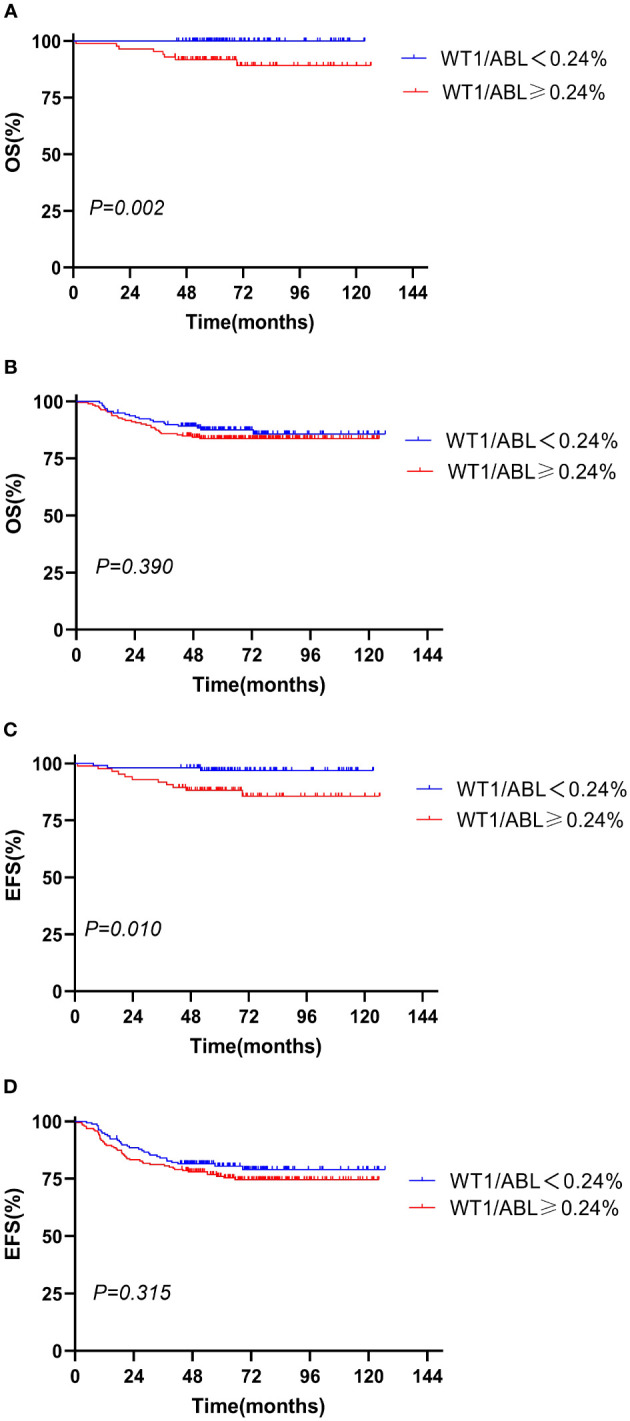
Overall survival and event-free survival according to the WT1 expression and risk stratification. **(A, C)** Standard risk; **(B, D)** intermediate risk and high risk.

**Figure 4:**
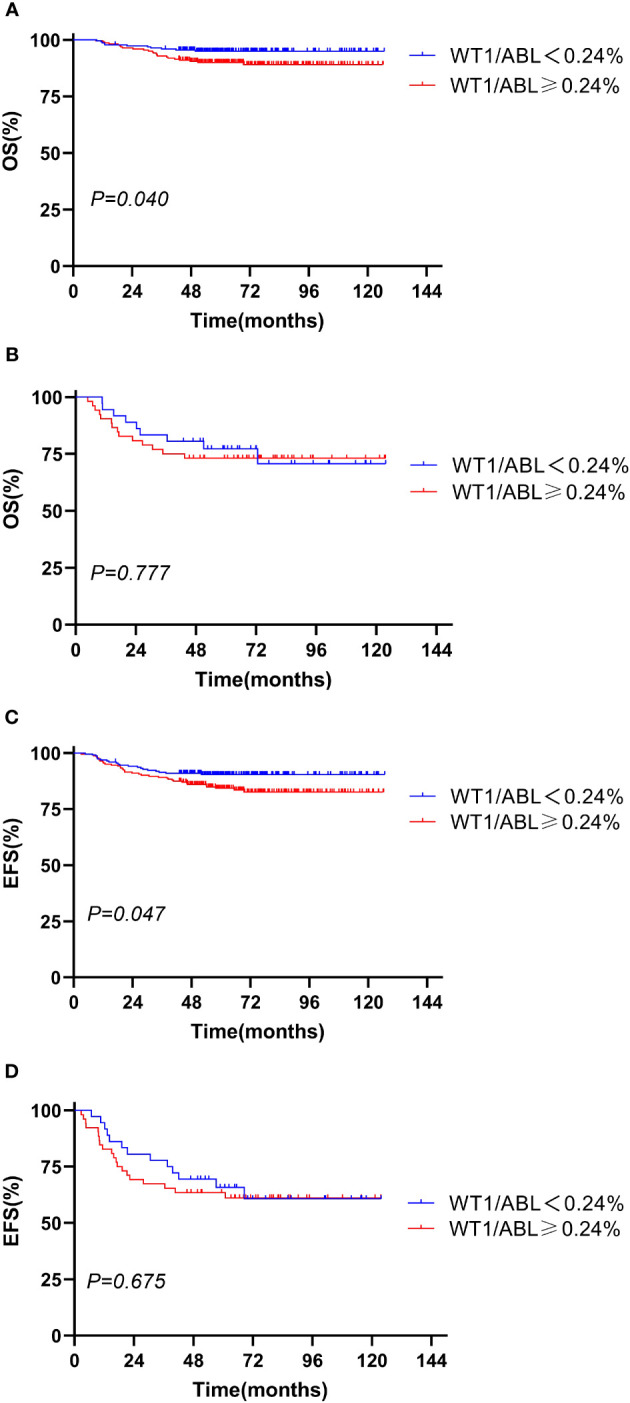
Overall survival and event-free survival according to the WT1 expression and day 33 minimal residual disease (MRD). **(A, C)** Day 33 MRD < 0.1%; **(B, D)** day 33 MRD ≥ 0.1%.

We evaluated the prognostic significance of WT1 expression in different clinical and treatment response subgroups (age, WBC, genotype, risk classification, day 33 MRD, and week 12 MRD). In patients with initial WBC<50 × 10^9^/L or in the standard-risk group or with MRD<0.1% at day 33, the OS and EFS rates tended to decline in the WT1 overexpression group compared with those in the WT1 downexpression group (*P*<0.05). Furthermore, patients with MRD<0.01% at week 12 had better EFS in the WT1 downexpression group than in the WT1 overexpression group (*P* = 0.034). As shown in **Supplementary Table S2**; **Figures 2**-**4**, the negative impact of WT1 overexpression was only observed in patients with initial WBC<50 × 10^9^/L or good response to treatment.

## Discussion

Currently, there are few studies on WT1 gene in pediatric BCP-ALL. We assessed the clinical specificity and prognostic value of WT1 expression in children with BCP-ALL in a comparatively large clinical data set and found a clear subgroup heterogeneity in the prognostic significance of WT1 gene.

In this study, children with WT1 overexpression had distinctive clinico-biological features, with WT1 overexpression taking place more frequently in the *KMT2A-r^+^* subgroup or the Pro-B subgroup or elder children. These findings are in consonance with the outcomes of previous studies (3, 4, 11). According to the study of Boublikova L et al. (11), WT1 overexpression tended to occur in pediatric ALL with *KMT2A-AFF1^+^* or initial age ≥10 years. Qin YZ et al. (3) and Wang S et al. (4) also reported that, in adult BCP-ALL, the WT1 transcript levels in the *KMT2A* rearrangement group were uniformly higher. However, a previous study with a small sample (*n* = 40) of pediatric ALL by Hagag A et al. (7) showed a remarkably older age in the negative WT1 gene expression group in comparison with the positive group, but with no essential differences between the two groups regarding other clinical presentations.

This discrepancy may be associated with influences—for example, the number of participants and the detection procedure of WT1 gene. In comparison with previous studies, we had the largest sample size that makes our outcomes more convincing. Moreover, previous research (3, 4) have displayed that RQ-PCR utilizing ABL as a control gene is a sensitive and reliable procedure for the quantitative detection of the WT1 gene.

In pediatric ALL, data on WT1 as an indicator of MRD monitoring are scarce and conflicting, and the prognostic role of WT1 also remains unclear. In our study, there was an escalated proportion of children obtaining a negative MRD at week 12 in the WT1 downexpression group that parallels with a significantly higher OS rate. These findings do not entirely coincide with previous studies. Zhang R et al. (12) detected a WT1-RNA transcript level during the induction and consolidation therapy in 107 ALL children to assess its reliability as an MRD monitoring index. However, the outcomes showed that the WT1 gene was not an excellent marker for MRD assessment in childhood ALL. Boublikova L et al. (11) assessed the WT1 expression in 125 childhood ALL and found that either increased or decreased WT1 expression suggested an escalated likelihood of recurrence. The justifications for the differences in outcomes may be associated with the composition of patients and the cutoff value of WT1 gene. The cases enrolled in our survey were uniform, and all children were BCP-ALL. Furthermore, the X-tile software has been reported to calculate the optimal cutoff point by utilizing the minimum probability value of the log-rank chi-square test (13). We found the optimal cutoff value of the WT1 gene for predicting prognosis as calculated by X-tile: 0.24%, which was more concrete and has not been reported in previous studies.

Moreover, the subgroup analysis showed that the poor prognostic significance of WT1 overexpression was more obvious in the standard-risk (SR) group with initial WBC<50 × 10^9^/L or good treatment response. This finding proposes for patients in the SR group, mainly for those with no particular risk-stratifying genes, that WT1 might be utilized as a continuously supervised biomarker to measure residual disease dynamics, just like MRD, consequently facilitating beforetime the identification of a high-risk subgroup. Unfortunately, several children in our cohort did not dynamically monitor the expression level of WT1 gene, so more subtle subgroup comparisons cannot be customized to further verify this conclusion.

WT1 is closely related to the occurrence, development, and prognosis of leukemia. However, its role in leukemogenesis has not been fully defined. The transcription factor encoded by WT1 gene is bidirectional, with the dual function of inhibiting tumor development and activating the transcription of oncogenes (14, 15). In leukemia, the overexpression of the WT1 gene has been reported to cause resistance of leukemic cells to chemotherapy (16–18), which can clarify the poor prognostic significance of WT1 overexpression in pediatric BCP-ALL in this survey.

## Conclusions

In conclusion, the cytogenetically defined pediatric BCP-ALL subgroups have unique WT1 expression patterns at initial diagnosis. WT1 overexpression predicted a poor outcome in B-other patients. The WT1 gene may serve as a biomarker for monitoring residual disease in the B-other population, especially in children in the SR group.

## Data availability statement

The original contributions presented in the study are included in the article/**Supplementary Material**. Further inquiries can be directed to the corresponding author.

## Ethics statement

The studies involving humans were approved by the Ethics Committee of Peking University People’s Hospital. The studies were conducted in accordance with the local legislation and institutional requirements. Written informed consent for participation in this study was provided by the participants’ legal guardians/next of kin.

## Author contributions

Y-jX: Writing – original draft. YW: Writing – original draft. L-pZ: Resources, Conceptualization, Writing – review & editing. A-dL: Data curation, Resources, Writing – review & editing. Y-pJ: Resources, Project administration, Supervision, Writing – review & editing. Y-xZ: Data curation, Resources, Writing – review & editing. H-mZ: Writing – review & editing, Project administration, Resources, Supervision.
