# Supplementary material for: Prognostic significance of Wilms’ tumor gene 1 expression in children with B-cell precursor acute lymphoblastic leukemia
Source: Front Oncol. 2024 Jan 15;13:1297870. doi: 10.3389/fonc.2023.1297870 (PMC10825953; doi:10.3389/fonc.2023.1297870)
Supplement: Supplementary file 1 [file DataSheet_1.docx]

1A


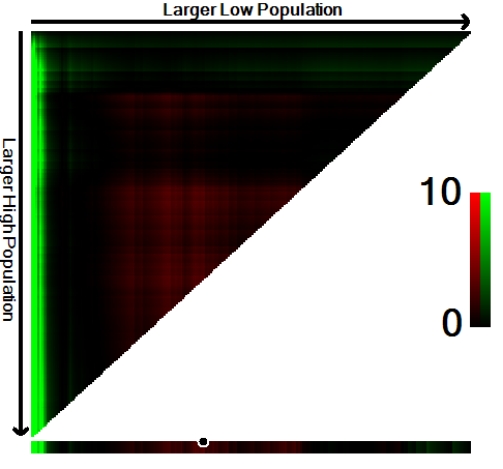


1B


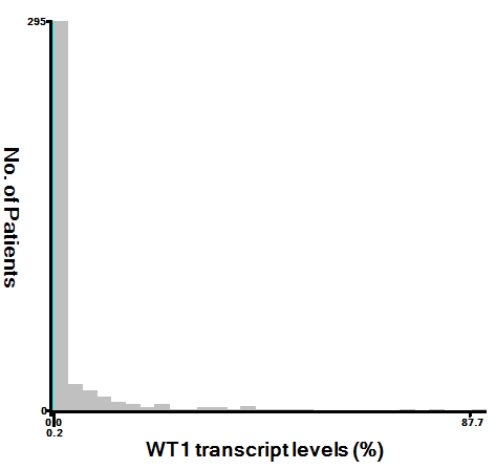


1C


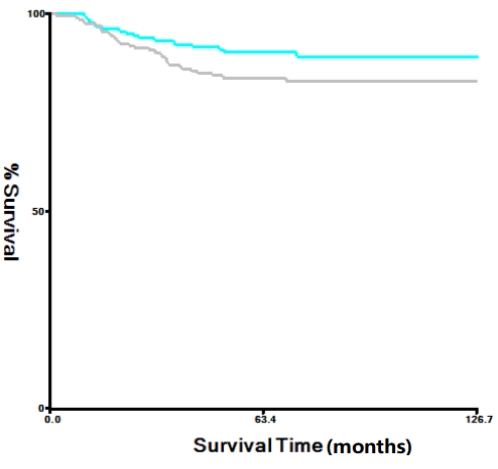


**Supplementary Figure 1.** X-tile (Yale University, New Haven, CT) analyses of survival data of children with BCP-ALL.

1A: X-tile plots were randomly divided into 2 groups according to WT1 transcript level. The x-axis of the X-tile plots represented all cut-off values from low to high (left–right), while the y-axis represented cut-off values from high to low (top–bottom). Red was associated with adverse survival, while green indicated direct associations. The optimal cut-off value for WT1 transcript level (0.24%), indicated by black/white circle is shown on x-axis.

1B: Different WT1 transcript values and their corresponding number of cases are presented on the histogram of the cohort.

1C: Kaplan-Meier plot estimate the survivals of patients according to the optimal cut-off value for WT1 transcript level (0.24%).

**Supplementary Table 1** Univariate and multivariate analysis of risk factors for overall survival and event-free survival in the BCP-ALL patients.

| Variable | OS | | EFS | |
| --- | --- | --- | --- | --- |
|  | Univariate(*P*) | Multivariate(*P*)  HR(95%CI) | Univariate(*P*) | Multivariate(*P*)  HR(95%CI) |
| Gender | 0.891 |  | 0.127 |  |
| Age (≥10) y | 0.033 | 0.297  1.340(0.773-2.323) | 0.044 | 0.387  1.218(0.781-1.900) |
| WBC≥50×10^9^/L | 0.022 | 0.385  1.289(0.727-2.284) | 0.034 | 0.308  1.318(0.776-2.237) |
| Immunophenotype | 0.852 |  |  |  |
| WT1-overexpression | 0.008 | 0.095  1.692(0.912-3.135) | 0.021 | 0.342  1.241(0.795-1.939) |
| *TCF3-PBX1* | 0.096 |  | 0.285 |  |
| *KMT2A-r* | 0.378 |  | 0.633 |  |
| *BCR-ABL1* | 0.925 |  | 0.821 |  |
| *ETV6-RUNX1* | 0.003 | 0.055  0.142(0.019-1.038) | 0.005 | 0.068  0.426(0.170-1.064) |
| Day33 MRD≥0.1% | ＜0.001 | 0.042  1.977(1.023-3.819) | ＜0.001 | 0.039  1.782(1.030-3.081) |
| Week12 MRD≥0.01% | ＜0.001 | 0.007  2.746(1.320-5.709) | ＜0.001 | ＜0.001  3.097(1.680-5.710) |

MRD, minimal residual disease; WBC, white blood count; HR, hazards ratio; CI, confidence interval; EFS, event-free survival; OS, overall survival.

**Supplementary Table 2**  Prognostic analysis of WT1 in different subgroups.

| Variables | 5y-OS(%) | | *P* | 5y-EFS(%) | | *P* |
| --- | --- | --- | --- | --- | --- | --- |
|  | WT1/ABL＜0.24% | WT1/ABL≥0.24% |  | WT1/ABL＜0.24% | WT1/ABL≥0.24% |  |
| Age(years) |  |  |  |  |  |  |
| ＜10 | 93.1±1.8 | 88.8±2.4 | 0.146 | 87.6±2.3 | 82.3±2.9 | 0.133 |
| ≥10 | 89.4±4.5 | 81.2±4.0 | 0.184 | 84.0±5.2 | 74.9±4.4 | 0.173 |
| Initial WBC (×10^9^/L) |  |  |  |  |  |  |
| ＜50 | 94.5±1.5 | 86.6±2.2 | 0.004 | 89.3±2.1 | 79.6±2.7 | 0.004 |
| ≥50 | 75.9±7.9 | 83.3±5.8 | 0.469 | 66.4±9.6 | 80.6±6.2 | 0.305 |
| Risk stratification |  |  |  |  |  |  |
| SR | 100.0 | 94.1±1.5 | 0.002 | 96.9±1.8 | 88.2±3.5 | 0.010 |
| IR | 92.0±2.6 | 87.5±2.9 | 0.199 | 85.3±3.3 | 79.2±3.7 | 0.144 |
| HR | 73.0±7.3 | 76.0±5.4 | 0.511 | 64.9±7.8 | 69.8±5.8 | 0.491 |
| Genotypes |  |  |  |  |  |  |
| *ETV6-RUNX1* | 97.9±2.1 | 100.0 | 0.381 | 93.6±3.6 | 93.3±4.6 | 0.820 |
| *TCF3-PBX1* | 100.0 | 100.0 | - | 93.8±6.1 | 85.7±13.2 | 0.736 |
| *KMT2A-r* | 75.0±21.7 | 85.7±7.6 | 0.620 | 75.0±21.7 | 81.0±8.6 | 0.786 |
| *BCR-ABL1* | 92.3±7.4 | 85.7±9.4 | 0.625 | 84.6±10.0 | 85.7±9.4 | 0.873 |
| Day 33 MRD |  |  |  |  |  |  |
| ＜0.1% | 94.9±1.5 | 90.0±2.0 | 0.040 | 90.4±2.0 | 84.3±2.5 | 0.047 |
| ≥0.1% | 77.2±7.1 | 73.1±6.2 | 0.777 | 65.8±8.1 | 63.5±6.7 | 0.675 |
| Week 12 MRD |  |  |  |  |  |  |
| ＜0.01% | 94.1±1.5 | 89.7±1.9 | 0.083 | 90.0±1.9 | 83.6±2.4 | 0.034 |
| ≥0.01% | 61.5±13.5 | 63.3±8.8 | 0.990 | 25.6±13.8 | 53.3±9.1 | 0.390 |

MRD, minimal residual disease; WBC, white blood count; CR, complete remission; EFS, event-free survival; OS, overall survival; NA, not available.
